# Innate Immune Evasion of Alphaherpesvirus Tegument Proteins

**DOI:** 10.3389/fimmu.2019.02196

**Published:** 2019-09-13

**Authors:** Linjiang Yang, Mingshu Wang, Anchun Cheng, Qiao Yang, Ying Wu, Renyong Jia, Mafeng Liu, Dekang Zhu, Shun Chen, Shaqiu Zhang, Xinxin Zhao, Juan Huang, Yin Wang, Zhiwen Xu, Zhengli Chen, Ling Zhu, Qihui Luo, Yunya Liu, Yanling Yu, Ling Zhang, Bin Tian, Leichang Pan, Mujeeb Ur Rehman, Xiaoyue Chen

**Affiliations:** ^1^Institute of Preventive Veterinary Medicine, Sichuan Agricultural University, Chengdu, China; ^2^Key Laboratory of Animal Disease and Human Health of Sichuan Province, Sichuan Agricultural University, Chengdu, China; ^3^Avian Disease Research Center, College of Veterinary Medicine, Sichuan Agricultural University, Chengdu, China

**Keywords:** alphaherpesvirus, immune evasion, tegument protein, IFN, signaling pathway

## Abstract

Alphaherpesviruses are a large family of highly successful human and animal DNA viruses that can establish lifelong latent infection in neurons. All alphaherpesviruses have a protein-rich layer called the tegument that, connects the DNA-containing capsid to the envelope. Tegument proteins have a variety of functions, playing roles in viral entry, secondary envelopment, viral capsid nuclear transportation during infection, and immune evasion. Recently, many studies have made substantial breakthroughs in characterizing the innate immune evasion of tegument proteins. A wide range of antiviral tegument protein factors that control incoming infectious pathogens are induced by the type I interferon (IFN) signaling pathway and other innate immune responses. In this review, we discuss the immune evasion of tegument proteins with a focus on herpes simplex virus type I.

## Introduction

Herpesviruses are divided into three subfamilies, alpha-, beta- and gammaherpesviruses, all of which share a common viral morphology and approximately 40 conserved genes that are important for virus production. The alphaherpesvirus subfamily has a wide range of host (1). Herpes simplex virus 1 (HSV)-1, HSV-2, and varicella-zoster virus (VZV) belong to the human alphaherpesvirus subfamily, while veterinary alphaherpesviruses include bovine herpesvirus (BHV), pseudorabies virus (PRV), and waterfowl duck enteritis virus (DEV) (2).

Herpesviruses undergo two forms of replication, lytic replication, and latent infection. In the lytic replication cycle, the virus first enters a cell, and the viral DNA begins to replicate after the capsid DNA is released into the nucleus. Subsequently, after assembly and genome packaging, the capsid leaves the nucleus (3). The viral particles then undergo primary envelopment and de-envelopment at the nuclear envelope, with tegumentation and secondary envelopment occurring in the cytoplasm. Finally, the virions leave the host by exocytosis (Figure 2) (4–6). After some alphaherpesviruses replicate at the infection site, the nervous system is invaded by the fusion of some alphaherpesviruses with the neuronal membrane at the end of an axon. When alphaherpesvirus DNA enters ganglion cell nuclei, some viral particles immediately assemble into a chromatin structure, forming heterochromatin, and resulting in latent infection (7). Not all neuronal infections lead to chromatinization, and in some cases, neuronal infection leads to lytic replication. The occurrence of lytic replication may depend on both viral and cellular factors that are differentially expressed in distinct types of neurons. Epithelial cells are the primary sites for alphaherpesvirus infection and are typically asymptomatic. Infected humans or animals become carriers without symptoms, with the infection becoming detectable only when progeny viral particles intermittently leave the host cells through germination, exocytosis or induction of apoptosis, making herpesviruses difficult to monitor and control (3, 8).

During latent infection, the viral genome remains in the nucleus, wherein new viral particles accumulate due to periodic reactivation of the lytic replication cycle and are transported along axons into epithelial cells, resulting in symptomatic or asymptomatic shedding (1, 9–12). Lytic replication releases infectious particles that elicit a strong immune response, whereas in latent infection, viruses use various strategies to weaken the presentation of antigens and prolong the lifespans of host cells (3, 13). This approach is highly beneficial to the survival of viruses and the establishment of latent infections.

**Figure 1:**
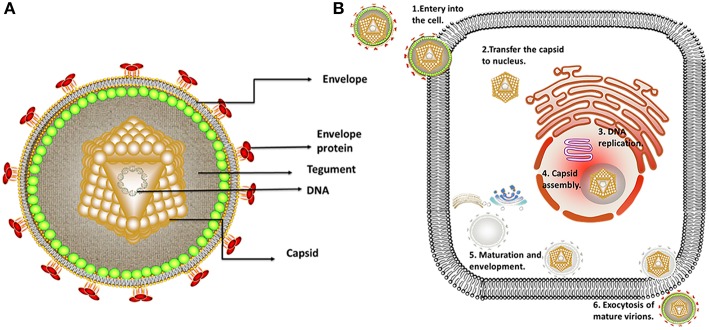
Structure and replication process of herpes virus. **(A)** Structure of alphaherpesviruses. The viral particle structure of alphaherpesviruses includes the genome, tegument, envelope, and capsid. **(B)** The viral replication process of alphaherpesviruses. The viral replication process of alphaherpesviruses includes adsorption, replication, and assembly, secondary envelopment and exocytosis. Some inspiration for this figure was obtained from previous articles (3).

Alphaherpesviruses encode ~8 capsid proteins, 23 tegument proteins (Table 1), and 14 envelope proteins (14, 15). The tegument is located between the capsid and the envelope (Figure 1). The alphaherpesvirus tegument is a self-supporting structure consisting of thousands of densely packaged protein molecules. A proteomic analysis of extracellular HSV-1 by mass spectrometry identified 23 types of virus-encoded tegument proteins as well as some host cell enzymes, chaperones and structural proteins, some of which may be incorporated into the tegument (14). The density of the tegument at the icosahedral vertices of the HSV-1 capsid has been observed by cryo-electron microscopy (cryo-EM), which revealed C-capsid-specific and capsid-apex-specific components (16). More generally, in different subfamilies of herpesviruses, the components are referred to the capsid-associated tegument complex (CATC) (17). Tegument proteins are typically designated as internal or external tegument components depending on whether they preferentially bind to the capsid or viral membrane during entry and exit or on their fractionation behavior after virus decomposition with non-ionic detergents. Although the outer tegument appears to be amorphous, the inner layer has a partial icosahedral order because of its close relationship with the capsid (18). Tegument proteins promote viral replication by regulating genes transcription, halting cell protein synthesis, and destroying host innate immune responses. They can also provide scaffolds for viral particles assembly and create interaction networks to link viral capsids and envelope proteins (1, 19). In addition to the important role of some tegument proteins in viral particles for immune evasion, some other viral proteins are also important for their survival, as they prompte viral replication and participate in the viral immune process. For example, HSV-1 UL24 (20) has key roles in modulating innate immunity. However, this review focuses on the innate immune escape of tegument proteins. The mechanism by which tegument proteins facilitate innate immune evasion remains unclear.

**Table 1:** Alternative alphaherpesvirus tegument genes and their homologs.

**HSV-1/2**	**VZV**	**PRV**
**Tegument proteins involved in innate immune evasion**		
UL13 (VP18.8)	ORF47	UL13 (VP18.8)
UL36 (VP1-2)	ORF22 (p22)	UL36
UL37	ORF21	UL37
UL41 (VHS)	ORF17	UL41
UL48 (VP16)	ORF10	UL48
UL49 (VP22)	ORF9	UL49
UL50 (dUTPase)	ORF8	UL50
US3	ORF66	US3
US10	ORF64/69	/
US11	/	/
RL1 (ICP34.5)	/	/
RL2 (ICP0)	ORF61	EP0 (ICP0)
RS1 (ICP4)	ORF62/71 (IE62)	IE180 (ICP4)
UL54 (ICP27)	IE63	UL54 (ICP27)
**Other tegument proteins**		
UL7	ORF53	UL7
UL11	ORF49	UL11
UL14	ORF46	UL14
UL16	ORF44	UL16
UL21	ORF38	UL21
UL23	ORF36	TK
UL47 (VP13-14)	ORF11	UL47
UL51	ORF7	UL51
UL55	ORF3	/
US2	/	/

Pattern recognition receptors (PRRs) are recognition molecules that are primarily expressed on the surface and in the intracellular compartments of innate immune cells. PRRs can recognize one or more pathogen-associated molecular patterns (PAMPs). The type I interferon (IFN) signaling pathway plays an important role in the innate immune response and is the first line of host defense against viruses (21). Among PRRs, Toll-like receptors (TLRs) were the first PAMP-detecting receptors to be discovered. Nucleic acids are detected by TLR3, TLR7, TLR8, and TLR9, which locate on the endosomal membrane (22, 23). TLRs detect PAMPs and subsequently recruit downstream binding proteins, such as bone marrow differentiation primary response protein 88 (MyD88), MyD88 binding protein-like protein (Mal), Toll/interleukin (IL)-1 receptor domain-containing adapter protein (TIRAP), and Toll/interleukin, which play important roles in the immune processes of HSV-1 infection. TLR3 can be activated by recognizing short double-stranded (dsRNA) and then further recruits and activates the adapter protein Toll/IL-1 receptor (TIR) domain-containing adaptor TRIF. Stimulation of the TLR3-TRIF signaling pathway activates the transcription factors NF-κB and IFN regulator factor 3/7 (IRF3/7), resulting in the translocation of NF-κB and IRF3/7 into the nucleus and the production of various cytokines, such as type I IFN (24). According to previous studies, HSV-1 can be detected by TLR2, TLR3, TLR4, and TLR9 (25). The cellular recognition of dsRNA or 5′-triphosphate dsRNA activates the expression of the retinoic acid-induced gene I (RIG-I) and melanoma differentiation-associated gene (MDA-5), resulting in homo-oligomerization of the mitochondrial antiviral signaling (MAVS) protein and activation of tank-binding kinase 1 (TBK1). In recent years, DNA sensors capable of detecting cytoplasmic DNA have been identified, including cyclic GMP-AMP (CGAMP) synthase (cGAS), IFN-γ inducible protein 16 (IFI16), DEAD-box polypeptide 41 (DDX41), DNA-dependent activator of IRF (DAI), and several proteins involved in the DNA damage response (DDR) (21, 26). Bacterial DNA, viral DNA, synthetic double-stranded DNA (dsDNA) and even dsDNA isolated from mammalian cells can be sensed in the cytosol if their lengths exceed 40–50 bp. The key DNA sensor cGAS, which binds to dsDNA and catalyzes the production of the second messenger 2′3′- cGAMP. cGAMP then binds to the binding protein stimulator of the IFN gene (STING), causing a conformational change in the dimerization of STING. TBK1 phosphorylates the serine at position 366 of STING and then recruits IRF3 (27). In addition to the DNA sensor cGAS, RNA polymerase III (POL III) also functions as a DNA sensor, and cytosolic POL III acts as an innate PRR that recognizes abundant foreign DNA in the cytosol. POL III transcribes the exogenous AT-rich DNA into 5′-ppp RNA, which is recognized by the cytoplasmic RNA sensor RIG-I, thereby allowing downstream signaling via the adaptor MAVS to activate NF-κB and IRF3. The activation of these proteins finally initiates host innate immune responses, including IFNs and proinflammatory cytokines (28–30). The binding of secretory IFNs to the homologous dimer receptors type I IFN receptor (IFNAR1) and type II IFN receptor (IFNAR2) induces the downstream Janus kinase (JAK)-signal transducer and activator of transcription factor (STAT) signaling pathway and antiviral IFN-stimulating gene (ISG) transcription (24).

Human and mouse genetic studies have found that type I IFN responses play an important role in controlling host innate immune responses to alphaherpesvirus infection. Human with mutations in STAT1, TLR3, or UNC-93B, whose gene products are involved in the production or responses of type I IFNs, are susceptible to HSV-induced encephalitis (31–34). Mouse models have demonstrated that type I IFNs are important for controlling acute alphaherpesvirus infection, and many gene products encoded by HSV can antagonize host type I IFN antiviral activity (35). Additionally, ISGs, such as ISG15 and 2′-5′-oligonucleotide synthase (OAS1), have been shown to be important for controlling acute alphaherpesvirus infection in mice (36, 37). Defects in TLR3 increases susceptibility to HSV encephalitis, while impairment of POL III induces predisposition to VZV encephalitis. This specificity may due to the important role of TLR3 in recognizing HSV in the central nervous system, while POL III appears to be an important sensor for the AT-rich VZV genome (38). Carter-Timofte et al. identified mutations in the POL III gene, located in the subunits POLR3A and POLR3E, in two of eight patients by whole-exome sequencing. Functional analysis demonstrated impaired expression of antiviral and inflammatory cytokines in response to the POL III agonist Poly (dA: dT) and increased viral replication in patient cells compared to these features in controls (39).

In addition to the type I IFN signaling pathway, some other innate immune pathways are also involved (40, 41). Chromosome breaks at specific sites caused by HSV-1 infection interact with cellular pathways that identify and repair DNA damage, also known as the DDR. Studies have shown that the DDR plays an active role in antiviral activity (42, 43). Autophagy functions in regulating the activity of specific signals utilized by cells and can remove the threat of intracellular pathogens and prevent the damage or accumulation of long-lived and aggregation-prone proteins (44). Therefore, autophagy is an important aspect of innate immunity. Moreover, because viral infection also induces the formation of antiviral cytoplasmic granules known as stress granules (SGs), this process is closely associated with SG formation and type I IFN production (45).

### IFN Induction and IFN-Dependent Signaling Pathways

#### Tegument Proteins Inhibit the TLR Signaling Pathway

**Figure 2:**
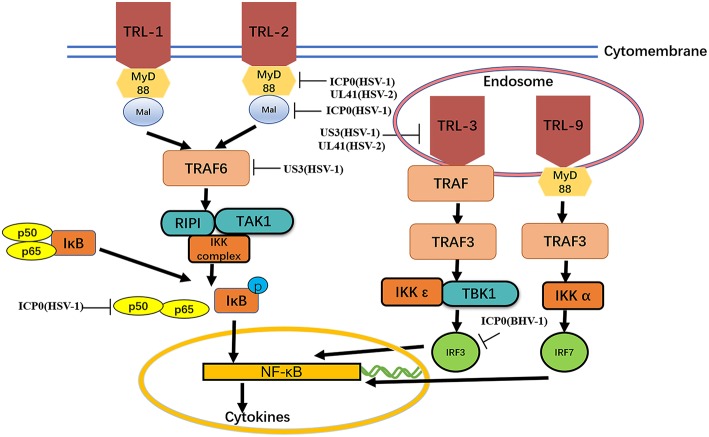
MyD88, Mal, TIRAP,TIRAP-induced IFN-β and TRAM. The formation of protein complexes of unique TBK1 and IKK inhibitors leads to activation of the transcription factors IRF3 and IRF7 and induction of IFN-β expression. Viral proteins can degrade TLRs and interfere with TLR recognition. The ubiquitination activity of viral proteins can inhibit MyD88, Mal, and TRAF6. A series of strategies is used for virus immune evasion. Some inspiration for this figure was obtained from previous articles (4).

TLRs are type I transmembrane protein that recognize microorganisms invading the body and activate immune responses (46, 47) and are thus believed to play a key role in the innate immune system. Downstream binding proteins of the TLR signaling pathway include MyD88, Mal, TIRAP, TRIF, and TRAM. TBK1 is ubiquitinated and autophosphorylated, leading to activation of the transcription factors IRF3 and IRF7 and induction of IFN-β expression (23) (Figure 2 and Table 2).

**Table 2:** Tegument proteins that inhibit the TLR pathway.

**Protein**	**Virus**	**Function**	**References**
RL2 (ICP0)	HSV-1	Reduces the inflammatory response triggered by TLR2	(48)
		Decreases MyD88 and Mal	(49)
US3	HSV-1	Reduces the levels of TLR3 and type I IFNs	(50)
		Inhibits TLR2 signaling by reducing TRAF6 polyubiquitination	(51)
UL41	HSV-2	Reduces the expression of TLR2 and TLR3	(52)

##### TLR2

The TLR2-dependent induction of type I IFNs occurs only in response to viral ligands. TLR2 can directly or indirectly promotes the synthesis and release of proinflammatory factors and enhances antiviral activities. Studies have also shown that infected cell protein 0 (ICP0) reduces the inflammatory response triggered by TLR2 during HSV-1 infection (48, 53, 54). van Lint and colleagues elucidated a process in which ICP0 promoted the degradation of TLR adapter molecules and inhibited inflammatory responses. ICP0 reduced the TLR-2-mediated inflammatory response to HSV-1 infection, and ICP0 expression alone is sufficient to block the expression of TLR-2 in MyD88 adapter complexes through the E3 ligase function of ICP0 (55–57). Yao and Rosenthal found that the expression of TLR2 in VK2 epithelial cells transfected with the HSV-2 virion host shutoff (VHS) protein was reduced, consistent with the findings in HEK 293 cells (52).

##### TLR3

TLR2 has been reported to linked to the recognition of several DNA viruses, while dsRNA is a particularly potent nucleic acid intermediate that activates TLR3 (58). TLR3 is capable of inducing the expression of type I IFNs and inflammatory cytokines after detecting the dsRNA. TLR3-deficient fibroblasts produced much less type I IFN during HSV-1 infection than the control group, and impaired TLR3 signaling also resulted in high level of viral replication (59). Cellular proteasomal activity is required for this inhibitory activity. Peri and colleagues observed that pUS3 interferes with TLR3 recognition and MxA induction following inhibition of type I IFN mRNA in HSV-1 infected cells (50). Similarly, Yao and Rosenthal found that the expression of TLR3 in VK2 epithelial cells transfected with the VHS protein was reduced, consistent with the findings in HEK 293 cells (52).

##### MyD88 and mal

MyD88 is an essential adapter molecule associated with inflammatory cytokines upon activation of all TLRs. The cascade pathway activates the transcription factor NF-κB and promotes the production of the proinflammatory cytokines IL-1β, IL-6, IL-8, IL-12, and monocyte chemotactic peptide 1 (MCP-1). Another MyD88-like protein, Mal, activates NF-kB, Jun amino-terminal kinase (JNK) and extracellular signal-regulated kinase-1 and−2. Mal can form homo- and heterodimers with MyD88 (60). van Lint showed that ICP0 can also decrease the level of MyD88 and Mal through its E3 ligase function (49).

##### TRAF6

The E3 ubiquitin ligase TNF receptor-associated factor 6 (TRAF6) interacts with TGF-β-activated kinase 1 (TAK1), subsequently activatingTAK1 (61). This interaction leads to activation of the IKK complex, which then phosphorylates the inhibitor of κB, causing κB ubiquitination and degradation (62). The HSV-1 kinase pUS3 can inhibit the TLR-2 signaling pathway by reducing TRAF6 polyubiquitination, which depends on its kinase activity before or at the stage of TRAF6 ubiquitination (51, 63).

#### Tegument Proteins Inhibit the RIG-I Signaling Pathway

**Figure 3:**
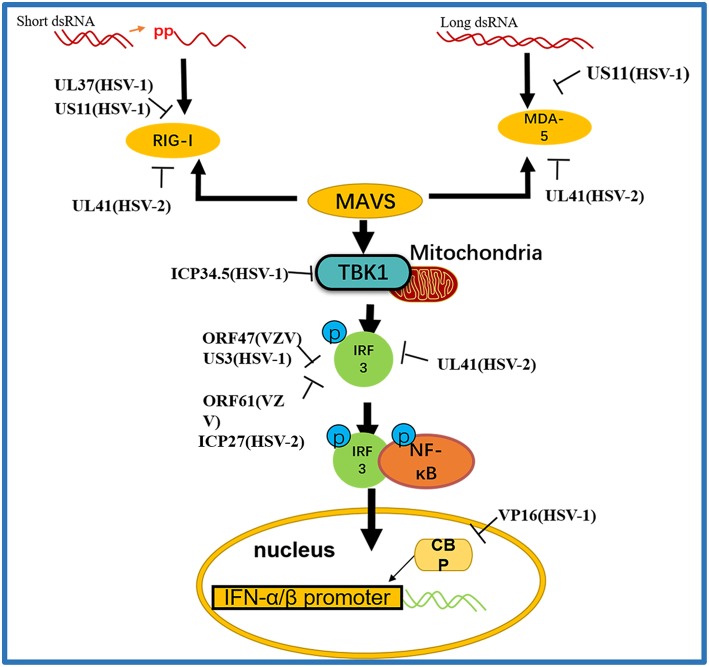
A schematic diagram of the pathogen-derived molecules used to escape intracellular RNA sensing pathways. The sensors in the pathway include RIG-I and MDA-5, which can detect different RNA species, primarily those containing 5′ diphosphate or triphosphate or long dsRNA, respectively. Pathogen-derived degradative or inhibitory helper proteins inhibit RIG-I activation through direct binding to block the interaction between RIG-I and MAVS and prevent RIG-I from entering mitochondria.

RIG-I and MDA-5 are members of the RIG-I-like receptor (RLR) family (64, 65) and can identify RNA viruses in cells and induce production of type I IFNs and immune factors (66). RIG-I activates NF-κB and IRFs through MAVS (67). Kato's gene knockout experiments showed that loss of RIG-I or MAVS could severely inhibit the innate immune response of mice, resulting in highly increased viral replication (68) (Figure 3 and Table 3).

**Table 3:** Tegument proteins that inhibit the RIG-I pathway.

**Protein**	**Virus**	**Function**	**References**
US3	HSV-1	Interacts with and hyperphosphorylates IRF3 to prevent IRF3 activation	(69)
US11	HSV-1	Binds to RIG-I and MDA5 inhibits their downstream signaling pathway	(70)
UL36 (VP1-2)	HSV-1	Deubiquitinates TRAF3 to prevent the recruitment of TBK1	(71)
UL37	HSV-1	Blocks RNA-induced activation by targeting RIG-I	(67)
RL1 (ICP34.5)	HSV-1	Binds to and sequesters TBK1	(72)
		Controls IRF3 activation by reversing translational shutoff and sustaining the expression of other IFN inhibitors	(73)
UL46	HSV-1	Blocks the interaction of TBK1 and IRF3 and inhibits the dimerization of TBK1	(74)
UL48 (VP16)	HSV-1	Blocks MAVS-Pex-mediated early ISG production	(75)
ORF61	VZV	Degrades activated IRF3	(76)
ORF47	VZV	Prevents IRF3 homodimerization and subsequent induction of IFN-β and ISG15	(77)
UL41	HSV-2	Inhibits RIG-I and MDA-5 as well as IRF3 dimerization and translocation	(52)
ORF62(IE62)	VZV	Blocks the phosphorylation of serine residues 396, 398, and 402 in IRF3	(78)
UL54(ICP27)	HSV-2	Inhibit IRF3 phosphorylation and nuclear translocation	(79)

##### RIG-I and MDA-5

RIG-I and MDA-5 act as two cytoplasmic dsRNA sensors. RIG-I primarily recognizes RNA containing 5′-triphosphate, while MDA-5 typically recognizes dsRNAs >2,000 bp in length. RIG-I and MDA-5 recruit MAVS to deliver signals to the kinase TBK1 and induce IκB kinase (IKKi), which phosphorylates both IRF3 and IκB kinase beta (IKKβ) and then activates the NF-κB signaling pathway (80). Once activated, IRF3 transfers to the nucleus and binds to positive regulatory domains I and III of the IFNβ promoter to induce IFNβ expression. Through coimmunoprecipitation analyses, Xing and colleagues demonstrated that in HSV-1 infected cells, the pUS11 C-terminus interacts with endogenous RIG-I and MDA-5 through an RNA binding domain. HSV-1 pUS11 can block IFN-β production and inhibit downstream signaling pathway activation by binding to RIG-I and MDA-5 (70). Zhao and colleagues observed that HSV-1 pUL37 is a deaminase protein that blocks RNA-induced activation by targeting RIG-I. Upon interacting with pUL37, RIG-I activation was inhibited (67). Yao and his colleague found that HSV-2 pUL41/VHS can inhibit the expression of RIG-I and MDA-5, thereby facilitating virus to evade host innate immune responses (52).

##### MAVS

Activated RIG-I and MDA-5 induce downstream signal transduction by binding to MAVS. The N-terminus of MAVS contains a CARD-like domain that binds to RIG-I and MDA-5, and through a CARD-CARD interaction to activate NF-κB and IRFs. MAVS is located in the outer mitochondrial membrane and interacts with RIG-I and MDA-5 to self-oligomerize (81). Peroxisome MAVS (MAVS-Pex) signaling has been reported to trigger the rapid production of IFN-dependent ISG in response to invasive pathogens (82). For example, pUL48/VP16, a tegument protein encoded by HSV-1, blocks the early production of ISG mediated by MAVS-Pex and inhibits the early innate immune signaling of peroxisomes (75).

##### TRAF3

TRAF3 is an important molecule in the RLR signaling pathway. The downstream kinases TBK1 and IκB kinase ε of RIG-I are recruited by the K63-mediated multiubiquitination of TRAF3, which results in IRF3 phosphorylation and the subsequent production of type I IFNs (83). pUL36 ubiquitin-specific protease has been shown to deubiquitinate TRAF3 and block the recruitment of the downstream adaptor TBK1 to decrease the production of IFN-γ during HSV-1 infection (71).

##### TBK1

As an IκB kinase-related kinase, TBK1 can phosphorylate a variety of substrates that are involved in various cellular processes (84). After DNA and RNA sensors detecting nucleic acids, TBK1 is activated. TBK1 triggers the phosphorylation of IRF3, the activation of NF-κB and the expression of type I IFNs. HSV-1 ICP34.5 is a neurotoxic factor with multiple functions and plays a crucial role in viral pathogenesis (85). Previous studies have reported that HSV-1 ICP34.5 can regulate IFN production by binding to and isolating TBK1 (72) and suppressing the induction of the ISG56 promoter by TBK1. Recently, study found that HSV-1 pUL46 interacted with TBK1 and reduced TBK1 activation and its downstream signaling. The results showed that pUL46 impaired the interaction between TBK1 and IRF3 and downregulated the activation of IRF3 by inhibiting the dimerization of TBK1 to reduce the production of type I IFN and immunostimulatory DNA (74).

##### IRF3

Activated IRF3 is essential for the effective transcription of type I IFNs, and IRF3 plays an important role in RLR-independent signal transduction. Activated IRF3 dimerizes and migrates to the nucleus, wherein it identifies specific sequence-based IFN stimulus response elements in the regulatory regions of target genes (86). Studies have shown that US3 protein expression can significantly inhibit the activation of IFN-γ, IFN stimulatory response element (ISRE) promoters and transcription of IFN, ISG54, and ISG56 via the neurovirus Sendai virus (SEV) (87). In addition, the SEV-induced dimerization and nuclear translocation of IRF3 have been shown to be blocked by pUS3. pUS3 can interact with and hyperphosphorylate IRF3 at serine 175, thus blocking IRF3 activation (69). Manivanh provided evidence that ICP34.5 controlled IRF3 activation via its ability to regulate translational shutoff reversal and by maintaining the expression of other IFN inhibitors encoded by viruses (73). The VZV immediate-early protein ORF61, a protein homologous to HSV-1 ICP0, attenuates the IRF3-mediated innate immune response through degradation of activated IRF3 (76). Vandevenne observed that during VZV infection, the VZV kinase ORF47, a protein homologous to UL13, can atypically inhibit the phosphorylation of IRF3, which blocks the homodimerization and induction of target genes such as IFN-β and ISG15 (77). VZV ORF62/IE62 is a protein homologous to HSV ICP4. Sen and colleagues found that the inhibition mediated by VZV IE62 may be the three serine residues (396, 398, and 402) on IRF3 were inhibited, thus blocking the downstream signal transduction mediated by IRF3 (78). Additional studies revealed that HSV-2 ICP27 directly associates with IRF3 and inhibits its phosphorylation and nuclear translocation, resulting in the inhibition of IFN-β induction (79) (Table 2).

#### Tegument Proteins Inhibit the NF-κB Signaling Pathway

The NF-κB signaling pathway is an important factor in antiviral immunity (88, 89) that promotes the expression of proteins contributing to viral replication and induces specific and adaptive immune responses (90). PPRs, TLRs, and RLRs can all lead to the induction of the NF-κB signaling pathway.

During HSV-1 infection, pUL48 can block IFN-β production by inhibiting NF-κB activation and interfering with the IRF3 recruitment of its coactivator CBP (91). The ORF61 protein of VZV and simian varicella virus (SVV) is involved in immune evasion and can prevent IκB-α from ubiquitination. Travis further demonstrated that SVV ORF61 can interact with β-TrCP, a subunit of the SCF ubiquitin ligase complex, to mediate the degradation of IκB-α (92). Sloan and colleagues observed that VZV ORF61 could inhibit the activity of the NF-κB reporter induced by tumor necrosis factor alpha (TNF-α). In addition, ORF61 mutation experiments revealed that the E3 ubiquitin ligase domain was necessary to inhibit the NF-κB pathway (93). During HSV-1 infection, ICP0 can interact with p65 and p50 and degrade the proteasomal protein p50 to block the nuclear translocation of p65 and reduce NF-κB-dependent genes expression (94). In contrast, another study showed that ICP0 can also ubiquitinate IκB-α and activate the transcription of NF-κB target genes (95). In addition, the replication of HSV-1 can be directly enhanced by stimulation of NF-κB, with recruitment of the ICP0 promoter by NF-κB activating the transcription and replication of ICP0 (96). The HSV-1 protein kinase US3 hyperphosphorylated p65 at serine 75 and blocked its nuclear translocation, significantly inhibiting NF-κB activation and decreasing the expression of the inflammatory chemokine IL-8 (97, 98) (Figure 2 and Table 4).

**Table 4:** Tegument proteins that inhibit the NF-κB pathway.

**Protein**	**Virus**	**Function**	**References**
UL48 (VP16)	HSV-1	Inhibits NF-κB activation and interferes with the IRF-3-mediated recruitment of its coactivator CBP	(91)
ORF61	SVV	Prevents IκBα ubiquitination and interacts with β-TrCP	(92)
	VZV	Inhibits TNF-α-induced NF-κB reporter activity	(93)
RL2 (ICP0)	HSV-1	Interacts with p50 and p60 and degrades the proteasomal protein p50	(94)
US3	HSV-1	Ubiquitinates IκBα	(95)
		Hyperphosphorylates p65 at serine 75 and blocks p65 nuclear translocation	(97)

#### Tegument Proteins Inhibit the DNA Sensor Signaling Pathway

**Figure 4:**
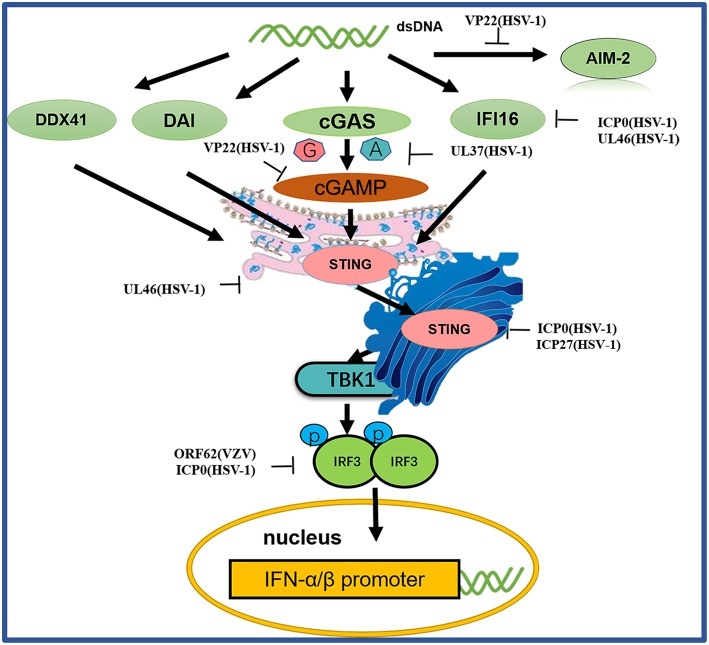
A schematic diagram of pathogen-derived molecules used to escape intracellular DNA sensing pathways. The primary sensor of cytoplasmic DNA is cGAS, which is responsible for activating the binding protein STING. Pathogen-mediated degradation targets cGAS to prevent it from binding to DNA or to inhibit its catalytic activity. At the same time, pathogen invaders also degrade cGAMP and bacterial circulating dinucleotides. IFI16 positively affects the activation of the cGAS-STING pathway. Other DNA sensors, such as DAI and AIM2, are also viral factors that block DNA binding and downstream pathway activation. Viral proteolytic enzymes can decompose and degrade these factors; blocking their translocation and preventing their interaction from the downstream signaling protein TBK1, thus hindering STING function.

In recent years, substantial advances have been made in research on DNA sensors. Several important cytoplasmic DNA sensors have been identified and characterized, providing insights into the mechanisms of sensor signaling pathways (21) (Figure 4 and Table 5).

**Table 5:** Tegument proteins that inhibit the DNA sensor signaling pathway.

**Protein**	**Virus**	**Function**	**References**
RL2 (ICP0)	HSV-1	Targets IFI16 degradation, inhibiting additional signaling and IRF-3 activation	(99)
		Blocks STING and the transcription factor IRF3	(100–102)
UL49 (VP22)	HSV-1	Inhibits the enzymatic activity of cGAS	(103)
UL46 (VP11-12)	HSV-1	Blocks STING and IFI16 transcript accumulation	(104)
UL37	HSV-1	Deamidates cGAS proteins	(105)
UL54 (ICP27)	HSV-1	Targets the TBK1-activated STING signalsome	(106)

##### cGAS

Among the DNA sensors, cGAS, a nucleotidyltransferase, is responsible for identifying various DNA ligands present in certain cell types. cGAS is activated by binding to cytosolic dsDNA and uses ATP and GTP to produce cGAMP through its enzymatic activity (107). Huang and colleagues showed that VP22 encoded by the HSV-1 UL49 gene is a tegument protein. VP22 participates in the innate immune antiviral process by inhibiting the enzymatic activity of cGAS and thus antagonizing the DNA-mediated innate immune signaling pathway (103). Zhang et al. discovered the innate immune evasion mechanism of the HSV-1 pUL37 deaminase protein and revealed that the human and mouse cGAS proteins (but not the nonhuman primate cGAS proteins) are targets for UL37 deamidation, promoting the lytic replication of HSV-1 (105).

##### STING

When STING is activated, it recruits TBK1, activates IRF3 and induces the production of IFN-β (103). As a broad antimicrobial factor, the DNA sensor STING antagonizes HSV by activating type I IFNs and proinflammatory responses upon sensing foreign DNA or non-canonical cyclic dinucleotides, the latter of which are synthesized by cGAS (108). Previous data suggested that ICP0 blocks the STING pathway (100). The transcription factor IRF3, a primary component of the STING pathway, is known to be blocked by ICP0 (101, 102), although the associated mechanism is unclear. Deschamps and colleagues showed that STING was degraded in cells expressing HSV-1 pUL46, which blocked the accumulation of STING transcripts (100). ICP27 interacted with TBK1 and STING in a manner that was dependent on TBK1 activity and the RGG motif in ICP27. Thus, HSV-1 inhibits the expression of type I IFNs in human macrophages through ICP27-dependent targeting of the TBK1-activated STING signalsome (106).

##### IFI16

IFI16, a member of the PYHIN protein family, was originally reported to be a cytosolic DNA sensor and has been implicated in the type I IFN response to HSV-1 (109–111). IFI16 localizes in the nuclei of many types of cells, making it a potential candidate for sensing HSV-1 DNA in the nucleus (112). Studies have shown that HSV ICP0 triggers IFI16 degradation, thereby inhibiting additional signaling and IRF3 activation (99, 113). Deschamps and colleagues also showed that IFI16 is degraded in cells constitutively expressing HSV-1 pUL46, which blocks the accumulation of IFI16 transcripts (104).

##### Other DNA sensors

Before the study of cGAS, proteins such as DAI, DDX41, DNA-dependent protein kinase (DNA-pk) and AIM2 were identified as cytosolic DNA sensor candidates (114). Although these proteins were reported to inhibit viral replication, further studies have shown that they are not necessarily involved in the DNA-induced responses in many human cells, suggesting that they may play a redundant or cell- type-specific role. HSV-1 AIM2-dependent inflammatory activation has been shown to be inhibited by the HSV-1 tegument protein VP22. VP22 can interact with AIM2 and prevent AIM2 oligomerization, which is the first step in AIM2 inflammasome activation (115).

#### Tegument Proteins Inhibit IFN-Stimulated Genes

**Figure 5:**
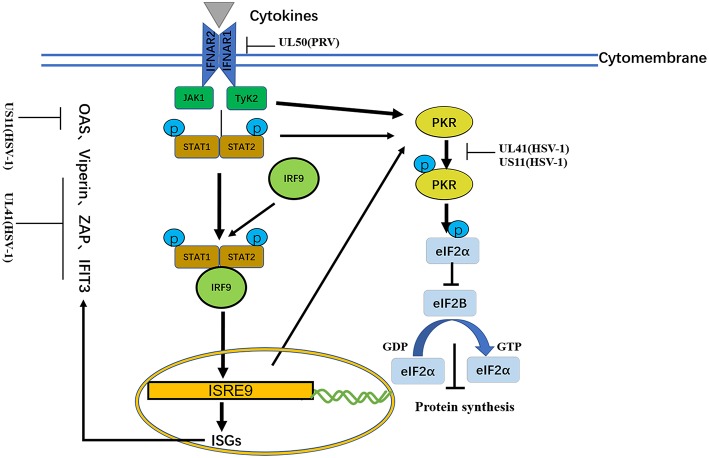
A schematic diagram of the pathogen-derived molecules used to escape cytokine-sensing pathways in cells. In the basic transmission process of the JAK/STAT signaling pathway, the binding of cytokines to their receptors causes dimerization of the receptor molecules. The close proximity, of JAKs to the receptors enables their activation through interactive tyrosine phosphorylation. The immune evasion effect of the virus can be achieved through the degradation of IFNAR1 and ISG mRNA.

Type I IFNs triggers numerous ISGs, such as viperin, zinc finger antiviral protein (ZAP), tetherin, dsRNA-dependent protein kinase (PKR), and OAS. Different combinations of ISGs can enhance the signaling transduction of type I IFNs and the antiviral activity of host to inhibit viral replication (21, 116) (Figure 5 and Table 6).

**Table 6:** Immune evasion of tegument proteins through ISGs.

**Protein**	**Virus**	**Function**	**References**
UL41	HSV-1	Degrades viperin mRNA	(117)
		Degrades hZAP mRNA	(118)
		Reduces the accumulation of IFIT3 mRNA	(119)
		Depletes tetherin mRNA	(120)
		Block the activation of PKR	(121)
US11	HSV-1	Inhibits OAS	(122)
		Inhibits PKR	(123)

##### Viperin

Viperin was first to be identified as a highly conserved protein that can induce IFN-γ protein production and is comprised of 361 amino acids. A number of studies have shown that viperin is directly induced by human cytomegalovirus and exhibits low expression (124). The viperin gene (also known as CIG5 or RASD2) can also be classified as an antiviral ISG that restricts the replication of DNA and RNA viruses (125). However, it is unclear whether viperin plays a role in HSV-1 infection. HSV-1 pUL41 can degrade host mRNA by cutting it at a preferential site, and UL41 promotes the replication of HSV-1 by degrading viperin mRNA (117).

##### ZAP

In addition to viperin, ZAP is an antiretroviral factor that was originally identified in rats. Viruses that contain ZAP response elements (ZREs) in their RNA are sensitive to ZAP. Studies have shown that human ZAP (hZAP) has no inhibitory effect on the replication of HSV-1, and as an antagonist of hZAP, HSV-1 pUL41/VHS can degrade hZAP mRNA (118).

##### Tetherin

Tetherin (BST-2 or CD317) is a membrane glycoprotein that can induce the production of IFNs and effectively exert antiviral activity by inhibiting the release of many envelope viruses (126). Helen showed that overexpression of tetherin can inhibit the replication of HSV-1 and that HSV-1 pUL41/VHS can deplete tetherin mRNA via its mRNA degradation function (120).

##### PKR

Binding of dsRNA activates PKR, which then phosphorylates the α subunit of eIF2α, resulting in translational inhibition (127). HSV-1 pUS11, a late-stage gene, inhibits PKR activation by binding to both dsRNA and PKR to prevent them from binding to each other (123), and then inhibits PKR phosphorylation. Other studies have shown that during early infection, the HSV-1 pUL41 VHS RNase protein degrades RNAs that activate PKR. The VHS RNase protein and mitogen-activated protein kinase act cooperatively to block the activation of PKR (121).

##### OAS

Similar to PKR, OAS recognizes dsRNA. The three primary forms of OAS recognize dsRNA through positively charged channels in the molecule. Conformational changes in OAS after binding to dsRNA lead to the synthesis of 2′,5′-oligoadenylates (2–5As), which then activate latent RNase L, leading to the degradation of viruses and endogenous RNA and the inhibition of viral replication. OAS is essential for host defense and can be inhibited by pUS11 via its dsRNA binding domain, with pUS11 sequestering any available dsRNA produced during infection as the potential underlying mechanism (122).

##### IFIT3

The IFN-induced protein with tetratricopeptide repeats (IFIT) family includes IFIT1 (ISG56), IFIT2 (ISG54), IFIT3 (ISG60), and IFIT5 (ISG58), which are distributed on human chromosome. Recent studies have shown that IFIT proteins restrict viral replication by altering protein synthesis, binding viral RNA or interacting with structural or non-structural viral proteins to exert antiviral effects (128). Jiang showed for the first time that IFIT3 has little effect on the replication of HSV-1, because pUL41/VHS reduces the accumulation of IFIT3 mRNA and disrupts its antiviral activity (119).

#### JAK/STAT Signaling Pathway

During viral infection, IFNs exert their antiviral function by inducing antiviral proteins via the JAK/STAT pathway (129). Four JAKs and seven STATs have been identified in mammals. JAKs are tyrosine kinases of the Janus family, include JAK-1, JAK-2, JAK-3, and Tyk-2. The STATs include STAT-1, STAT-2, STAT-3, STAT-4, STAT-5a, STAT-5b, and STAT6. JAK-1, JAK-2, Tyk-2, STAT-1, STAT-2, STAT-4, and STAT-5 are directly involved in IFN-mediated signaling transduction pathways. The JAK/STAT signaling pathway is a common pathway that includes many cytokine signaling molecules and plays extensive roles in cell proliferation, differentiation, apoptosis and inflammation. This pathway exerts its function by interacting with negative regulators in other signaling pathways and STAT-mediated covalent modifications. In the basic transmission process of the JAK/STAT signaling pathway, the binding of cytokines with their receptors induces receptor molecule dimerization. The proximity of JAKs to receptors then enables JAK activation through interactive tyrosine phosphorylation. Activated JAKs catalyze the tyrosine phosphorylation of receptors and form corresponding STAT docking sites, which enable STATs to bind to receptors through the SH2 domain and cooperate with JAKs. After phosphorylation, STATs form homodimers that divert into the nucleus, wherein they bind to the promoters of target genes to activate their transcription and expression (Figure 5 and Table 7).

**Table 7:** Immune evasion of tegument proteins through the JAK/ STAT signaling pathway.

**Protein**	**Virus**	**Function**	**Reference**
UL50	PRV	Promotes the lysosomal degradation of IFNAR1	(130)

##### IFNAR1

Because lacking intrinsic protein kinase domains, IFNAR1 and IFNAR2 rely on the 74 members of the JAK family for signal transduction (131). The results published by Zhang suggested that pUL50 has dUTPase activity. dUTPase catalyzes the hydrolysis of dUTP into dUMP and inorganic pyrophosphate, providing the dUMP precursor for dTTP biosynthesis and inhibiting IFN signaling. dUTPase also has the ability to suppress type I IFN signaling by promoting the lysosomal degradation of IFNAR1, thereby contributing to innate immune evasion (130) (Table 6).

#### Cytokine Signaling Pathways

Cytokines are small molecular proteins with extensive biological activity stimulated by immune cells (such as monocytes, macrophages, T cells, B cells, and natural killer (NK) cells) and some non-immune cells (endothelial cells, epidermal cells, and fibroblasts). Cytokines are produced by many kinds of cells induced by immunogens, mitogens or other stimulants and have many functions such as regulating innate and adaptive immunity, hematopoiesis, cell growth, and damaged tissue repair. Aside from IFNs, other cytokines can be classified into the following categories according to their function: ILs, TNF-α, TNF-β, colony-stimulating factors, chemokines, and growth factors (Table 8) (132).

**Table 8:** Immune evasion of tegument proteins through cytokine signaling.

**Protein**	**Virus**	**Function**	**References**
UL49 (VP22)	HSV-1	Interacts with AIM2 and prevents its oligomerization	(115)
		Inhibits OAS	
UL41	PRV	Reduces the expression of TNF-α	(133)
UL41	HSV-1	Suppresses cytokines such as IL-1β and IL-18	(134)
RL2 (ICP0)/RS1 (ICP4)	HSV	Downregulates SLPI or activates NF-κB	(135)
UL13	HSV-1	Induces SOCS1 and SOCS3	(136)
US10	DEV	Downregulates the transcript levels of IL-4, IL-6, and IL-10	(137)

#### SOCS1 and SOCS3

Suppressor of cytokine signaling 1 (SOCS1) and SOCS3 contain kinase inhibitory regions (KIRs) that can inhibit JAK signal transduction through the SH2 domain and interact with the phosphotyrosines of JAK and GP130, respectively (138). In addition to IFNs, PAMPs are effective inducers of SOCS1 and SOCS3. Because SOCS proteins negatively regulate cytokine signal transduction, many viruses induce the expression of SOCSs to aid in their survival (139). The SOCS family has eight members and suppresses various cytokine signaling pathways, including the IFN signaling pathway. In one study, the expression of eight SOCS family members during HSV-1 infection was analyzed by q RT-PCR, revealing that the tegument protein pUL13 could induce SOCS1 and SOCS3. However, no such induction was observed in UL13-deficient virus-infected cells, suggesting that the UL13 protein kinase was involved in the induction of the two genes (136).

#### TNF-α

TNF-α is a cytokine with multipotent biological effects that are triggered by two types of TNF-α receptors on the cell surface (140). The TNF-α signaling transduction pathway primarily involves caspase family-mediated apoptosis and activation of the transcription factors NF-κB and JNK protein kinase mediated by TRAF (141). The expression of TNF-α has been observed to be increased in the spleens of mice infected with PRV UL41 mutant virus. TNF-α is considered to be an important cytokine in innate immune responses. In addition, PRV UL41 plays an important role in targeting host innate immune responses via its ribonuclease activity. Studies have suggested that pUL41 may contribute to the protection of organisms from viral damage mediated by TNF-α via degradation TNF-α mRNA (133).

#### SLPI

Secretory leukocyte protease inhibitor (SLPI), an anti-inflammatory mediator of mucosal immunity, can inhibit both human immunodeficiency virus (HIV) and HSV in cell culture. Epidemiological studies have shown that high concentrations of SLPI in mucosal secretions can inhibit HIV transmission. Whether the loss of SLPI caused by HSV allows the virus to evade the host's innate immune response is currently being studied, and the loss of SLPI may lead to an increased risk of HIV infection in the context of HSV infection (142). Reverse transcription PCR experiments have shown that SLPI is lost due to downregulating genes expression. The downregulation of SLPI is related to NF-κB signaling pathway activation and inflammatory cytokine upregulation. Fakioglu showed that the ICP4- or ICP0-induced expression of immediate-early genes can downregulate SLPI or activate NF-κB (135).

#### ILs

ILs are cytokines that are produced by and used in many types of cells. Currently, at least 38 ILs, named IL-1 to -IL38, have been identified. These ILs have complex functions, forming networks and exhibiting complex overlaps, and playing important roles in the maturation, activation, proliferation, and immune regulation of immune cells. In addition, they also participate in various physiological and pathological reactions in organisms. For example, the proliferation, differentiation and functions of immune cells are regulated by a series of cytokines. According to their structure, cytokines can be divided into several protein families, such as the IL-1, IL-6, IL-10, TNF, and hematopoietic factor families (143). ILs can lead to local inflammation and cause sterilization and cell damage. Suzutani showed that a UL41-deleted strain of HSV-1 exhibited 20- and 5-fold higher sensitivity to IFN-α and IFN-β than the wild-type strain, respectively. These results indicate that one important role of HSV-1 pUL41/VHS *in vivo* is the evasion of non-specific host defense mechanisms during primary infection by suppressing cytokines such as IL-1β and IL-18 (134). A study by Ma (137) showed that DEV pUS10, which plays an important role in viral replication, could upregulate the transcription of IL-4, IL-6, and IL-10 in US10-deleted DEV-infected duck embryonic fibroblasts (DEFs) at all assayed time points (Table 7).

### NK Cells

Functional NK cells are essential for limiting herpesvirus transmission and disease symptoms. There are many types of receptors on the NK cell surface, and their functions can be divided into two categories: activation and inhibition of the proliferation system. By recognizing specific ligands, NK cells can sense changes of target cells surface properties. To prevent clearance from cytotoxic T lymphocytes, some viruses actively reduce the level of MHC class I (MHC-I), an important ligand of the KIR family on the cell surface inhibiting NK cell receptors (144). To benefit their survival, viruses can encode MHC-I-like proteins that activate KIR receptors and proteins that inhibit the exposure of NK cell receptor ligands.

CD300a, also known as IRP60, is a highly conserved inhibitory NK cell receptor that does not bind to MHC-I. CD300a is a 60 kDa protein belonging to the immunoglobulin (Ig) superfamily that is characterized by a single V-type Ig-like domain in its extracellular domain and several tyrosine-based immunoreceptor inhibition motifs (ITIMs) in its cytoplasmic domain (145). CD300a can identify aminophospholipids exposed on the cell surface, especially phosphatidylserine (PS) and phosphatidylethanolamine (PE), which can inhibit the cytolysis of NK cells by binding to ligands (146). CD300a inhibitory receptors and their lipid ligands have been specifically reported on mammals, birds and fish (147). To date, no descriptions of the NK cell evasion strategy involving CD300a have been reported. A study by Grauwet firstly indicated that the pUS3 protein kinase of the alphaherpesvirus PRV can trigger the inhibitory NK cell receptor CD300a binding to the surface of infected cells, thereby increasing the CD300a-mediated protection of the infected cells. In addition, the binding of pUS3 to CD300a is associated with the aminophospholipid ligand of CD300a and the IP21 activating kinase (148), thus representing a novel alphaherpesvirus strategy for escaping NK cells.

### Other Innate Immune Responses

#### The DDR Response

The cellular DDR pathway monitors damage to genomic DNA. DNA-PK, ataxia telangiectasia mutated (ATM) kinase, and ATM- and Rad3-related (ATR) kinase are the primary signaling pathway mediators that initiate the DDR (48). Recently, cellular DNA repair machinery was demonstrated to recognize viral genetic material (149). The DDR plays an important role in viral infection, participating in the activation of many components of the ATM-dependent signaling pathway and inhibiting the DNA PKC- and ATR-dependent arms (150). Lilley and colleagues showed that RNF8 and RNF168, important mediators of ATM-dependent signaling pathways, are targeted for proteasome-mediated degradation by ICP0 (151).

#### ER Stress

The endoplasmic reticulum (ER) is a cytoplasmic eukaryotic organelle that has numerous functions, taking part in the transport of cellular materials, the provision of increased surface areas for cellular reactions, and the production of proteins, steroids and lipids (152). Mis- and unfolded proteins that can cause stress in the ER accumulate during viral replication and trigger the unfolded protein response (UPR) (153). The IRE1/XBP1 pathway is the most conserved component of the UPR branch in eukaryotic cells (154). IRE1 is a dual-activity enzyme that contains a serine-threonine kinase domain and a ribonuclease domain (155). Upon activation, IRE1 undergoes dimerization and transphosphorylation, which facilitates the removal of a 26-nucleotide (NT) intron from the XBP1 gene to form a spliced XBP1, which translated into a transcription factor. In the nucleus, XBP1 induces the expression of the genes that enhance the folding ability of ER proteins and functions in phospholipid biosynthesis and ER-associated protein degradation (ERAD) (156). During HSV-1 infection, the molecular mechanism by which the IRE1/XBP1 branch of the UPR is repressed remains unclear. Zhang and colleagues showed that the HSV-1 tegument protein pUL41, which has endoribonuclease activity, degrades XBP1 mRNA to inhibit its expression (157). These findings reveal a novel mechanism by which HSV-1 modulates the IRE1/XBP1 branch of the UPR. Interestingly, the HSV-1 ICP0 promoter can react to ER stress. Burnett and colleagues found that ICP0 can activate itself independently during HSV-1 infection, suggesting that HSV-1 regulates the ER stress response through ICP0 (153).

## Conclusion

Over millions of years of coevolution between host and viruses, host species have developed a highly complex set of physiological immune mechanisms to block and eliminate viral infection. However, for every host immune response step, viruses have developed corresponding immune escape mechanisms to ensure their own survival. Successful immune escape is a primary factor underlying chronic herpesvirus infection. In recent years, substantial progress has been made in understanding the variety of cytoplasmic DNA sensors that enable resistance to the immune response. Tegument proteins play important role in alphaherpesvirus innate immune evasion. However, despite the mapping of antiviral defense signaling pathways between tegument proteins and host, a functional understanding of how tegument proteins work together to interfere with the innate immune system remains elusive. Further studies on the mechanisms of tegument proteins, capsid proteins, and glycoproteins will be helpful in the search for antiviral targets and development of antiviral drugs.

## Author Contributions

LY wrote the manuscript and constructed the figures. AC and MW contributed ideas for the review. QY, YWu, RJ, ML, DZ, SC, SZ, XZ, JH, YWa, ZX, ZC, LZhu, QL, YL, YY, LZha, BT, LP, MR, and XC edited and revised the manuscript.

### Conflict of Interest Statement

The authors declare that the research was conducted in the absence of any commercial or financial relationships that could be construed as a potential conflict of interest.

## Abbreviations

HSV-1: Herpes simplex virus 1
BHV: Bovine herpesvirus
PRV: Pseudorabies virus
DEV: Duck enteritis virus
PRRs: Pattern recognition receptors
PAMPs: Pathogen-associated molecular patterns
IFN: Interferon
TLRs: Toll-like receptors
VZV: Varicella zoster virus
RIG-I: Retinoic acid-inducible gene I
MDA5: Melanoma differentiation-associated gene 5
TBK1: Tank-binding kinase 1
cGAS: cyclic GMP-AMP synthase
DDR: DNA damage response
ISGs: IFN-stimulated genes
NF-κB: Nuclear factor kappa B
TNF-α: Tumor necrosis factor alpha
ZAP: Zinc finger antiviral protein
SVV: Simian varicella virus
SOCS: Suppressor of cytokine signaling
ATM: Ataxia telangiectasia mutated.
